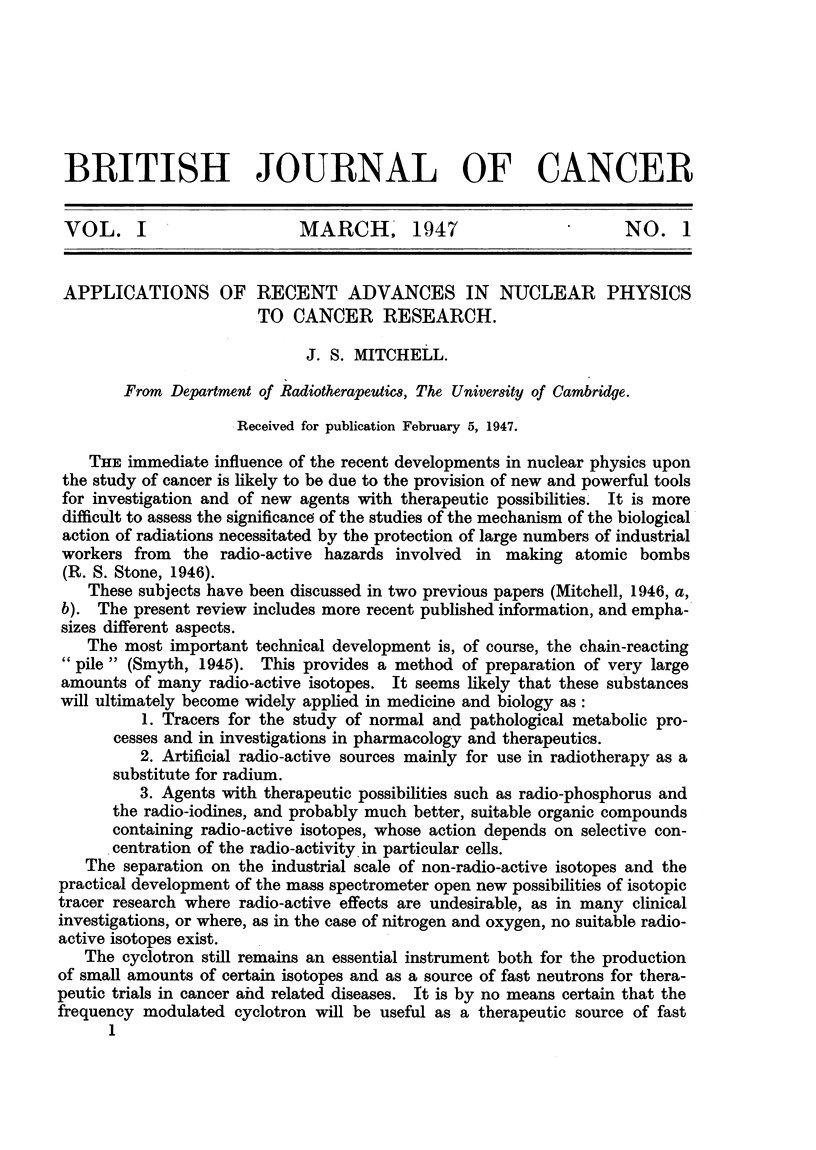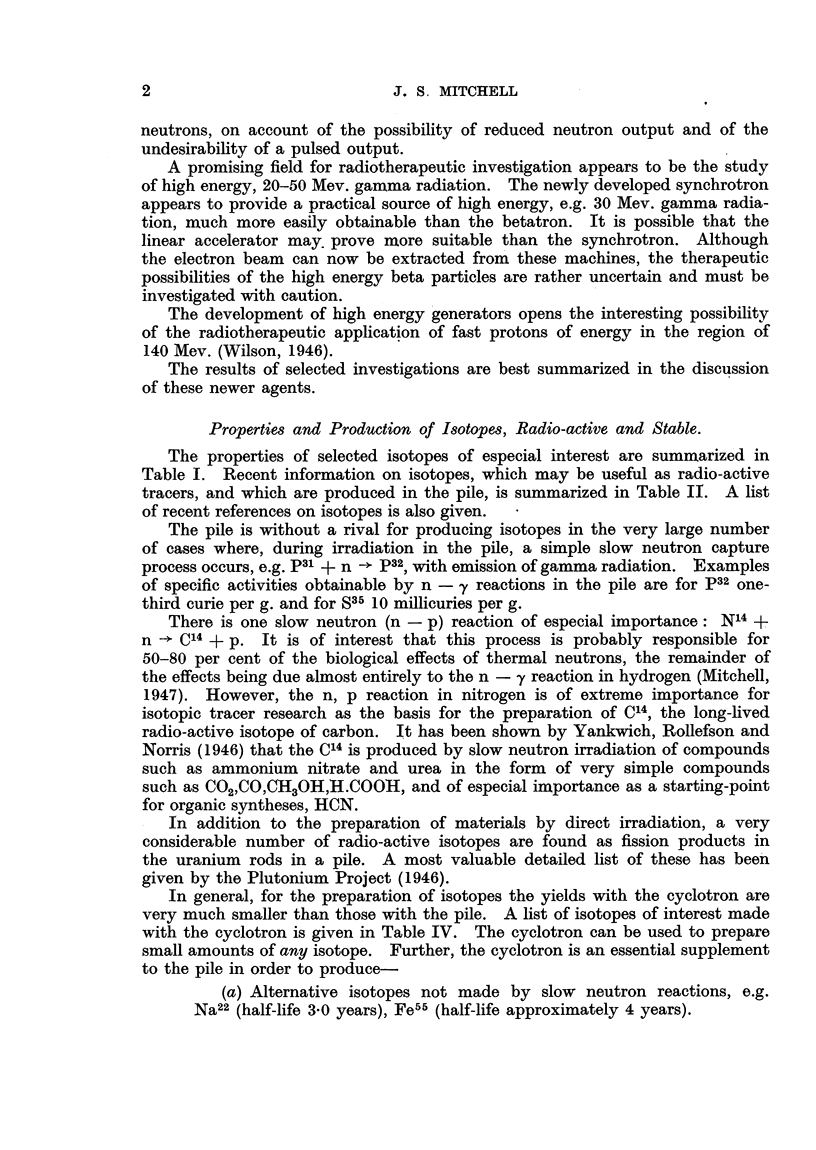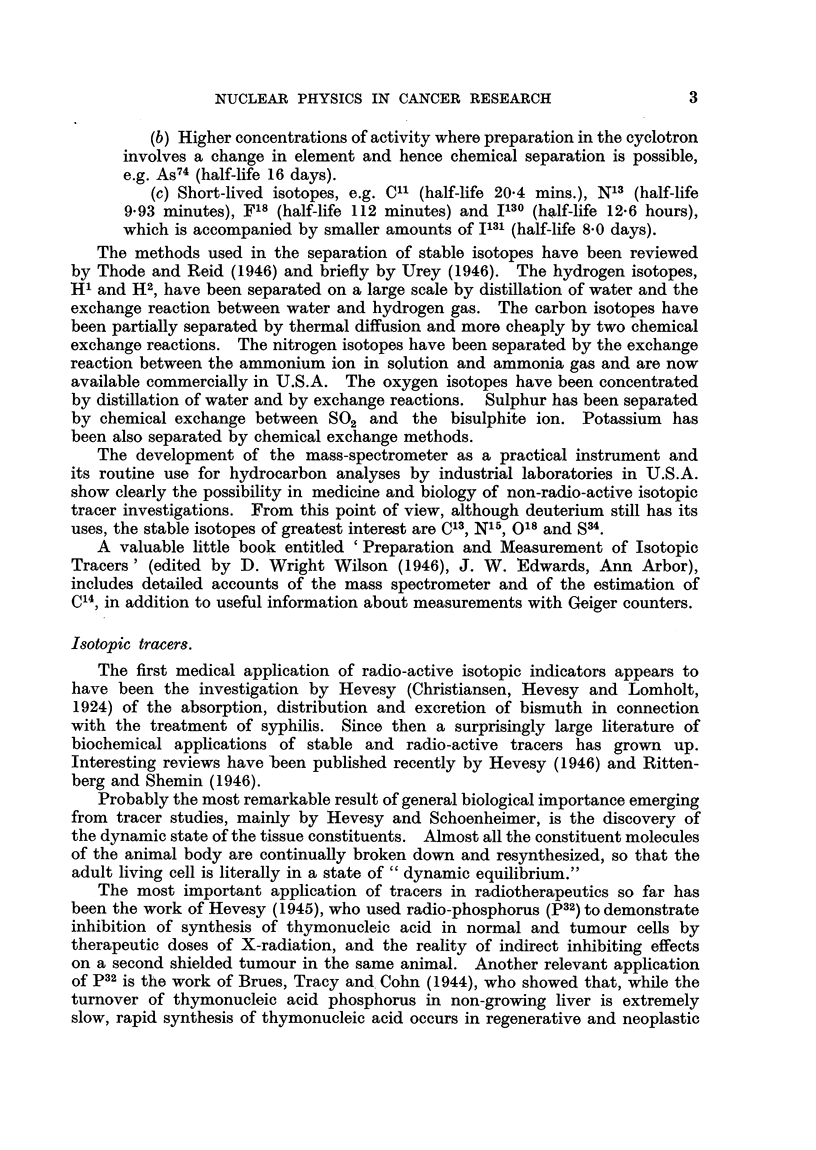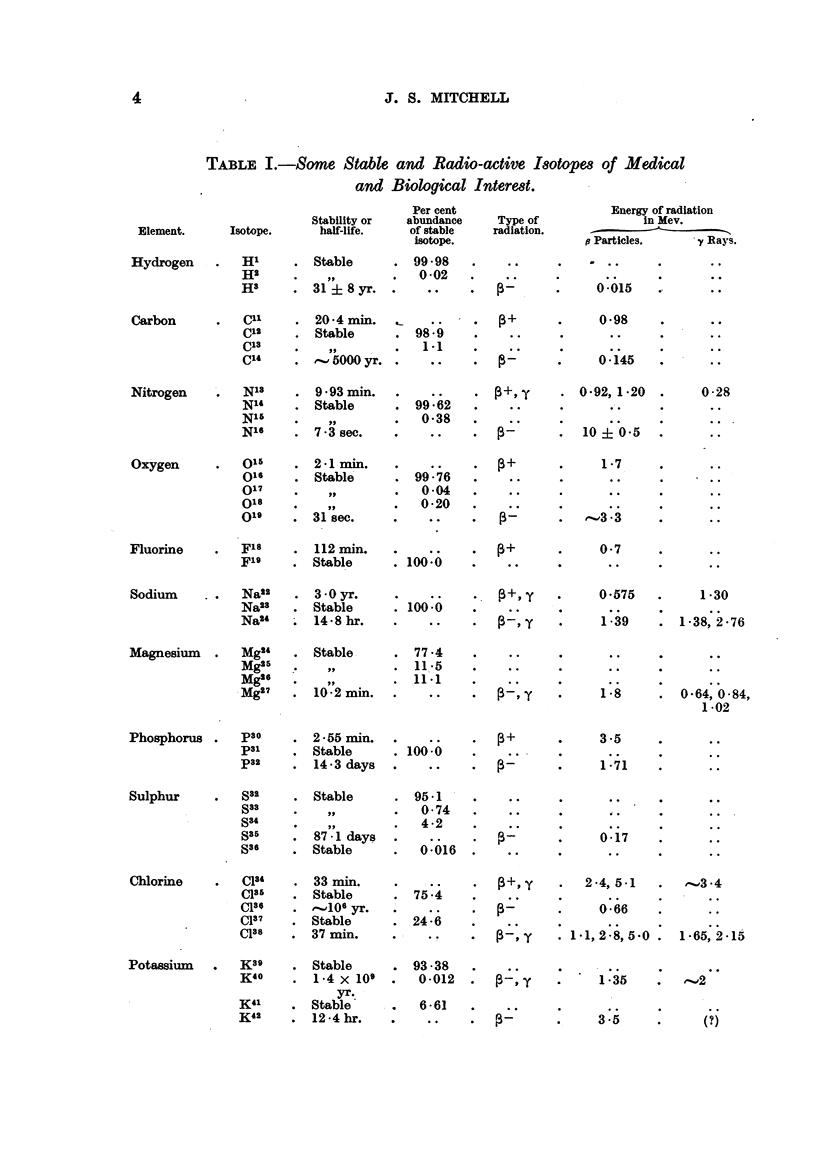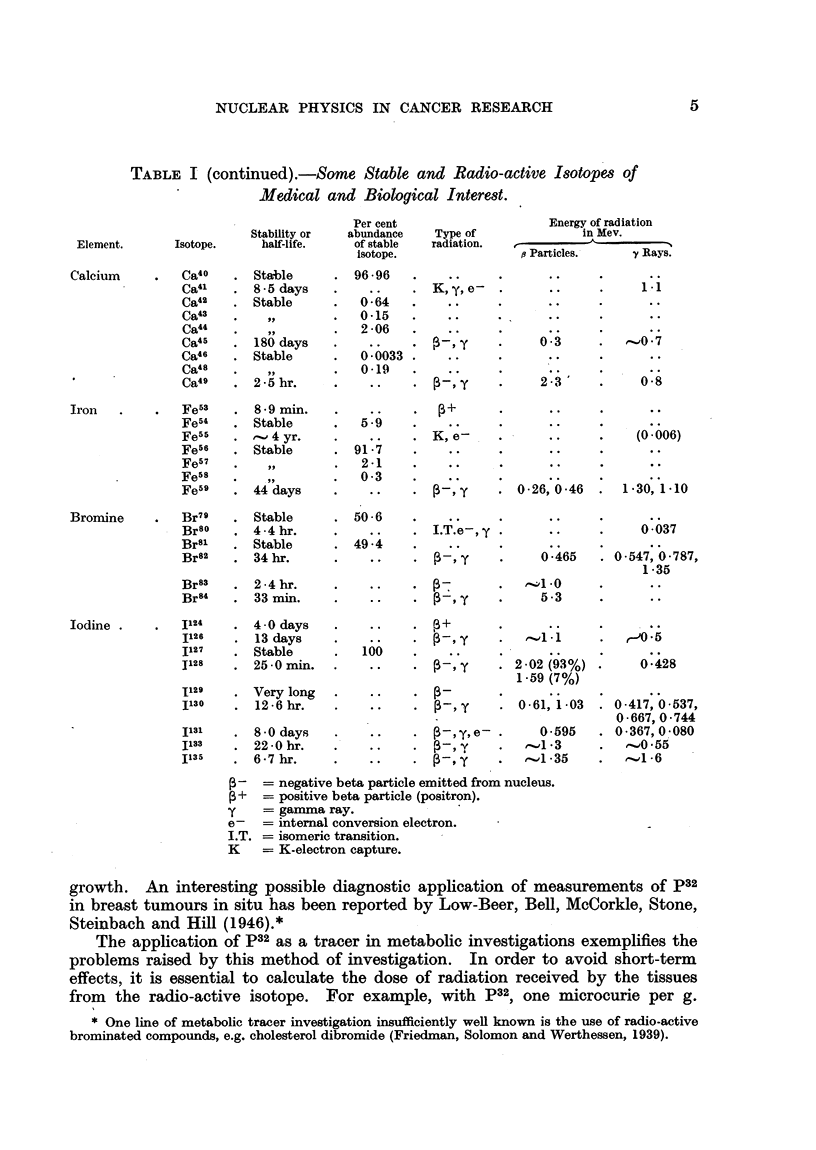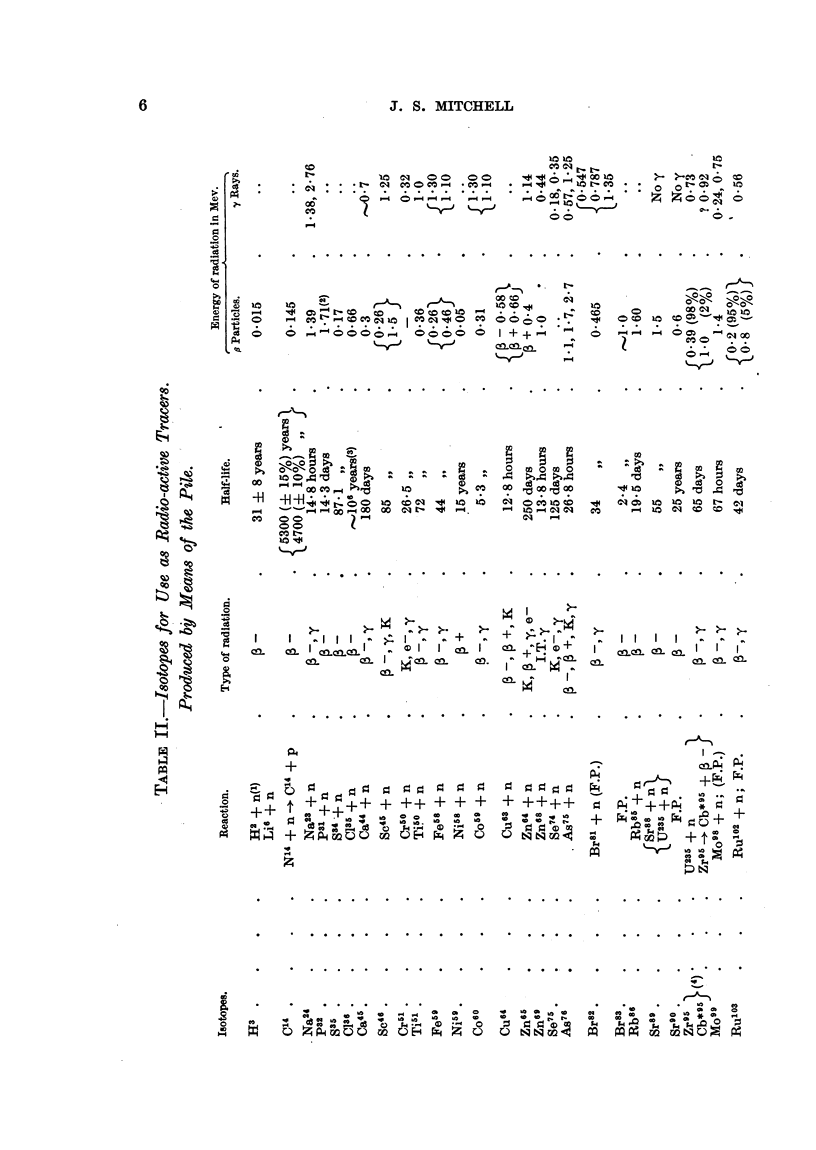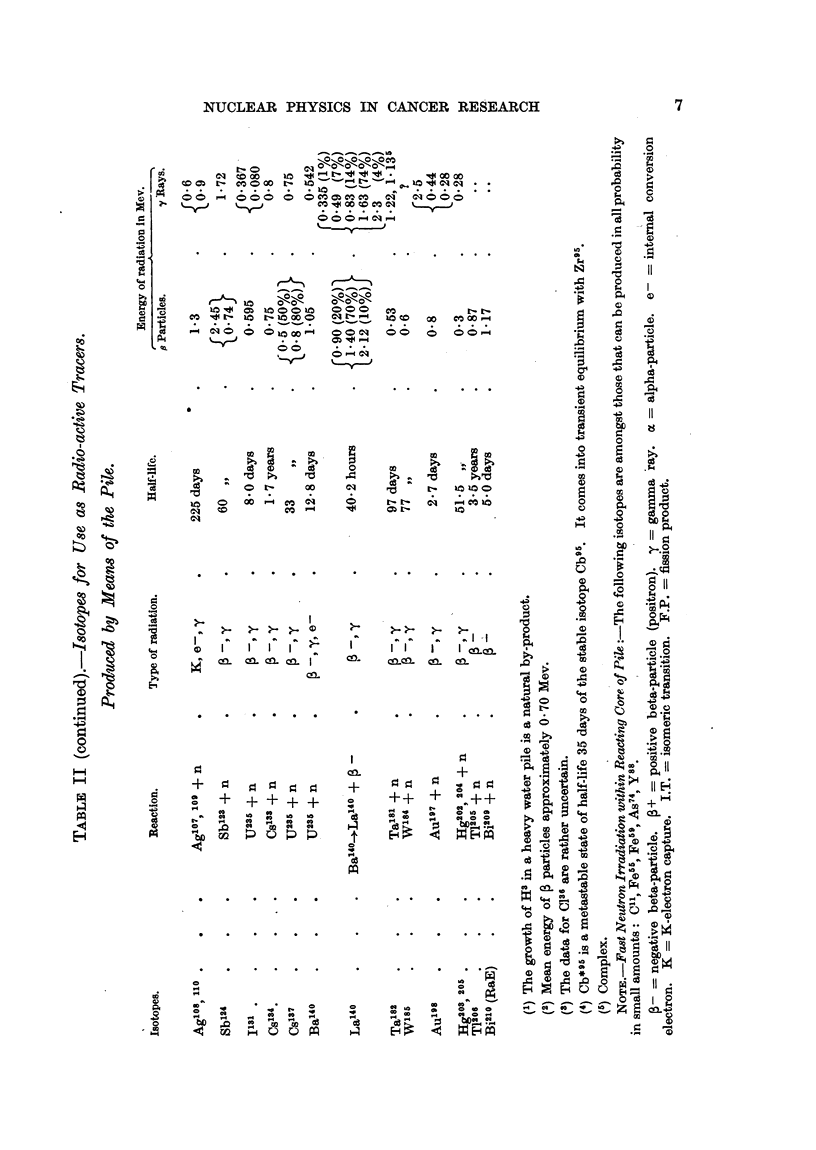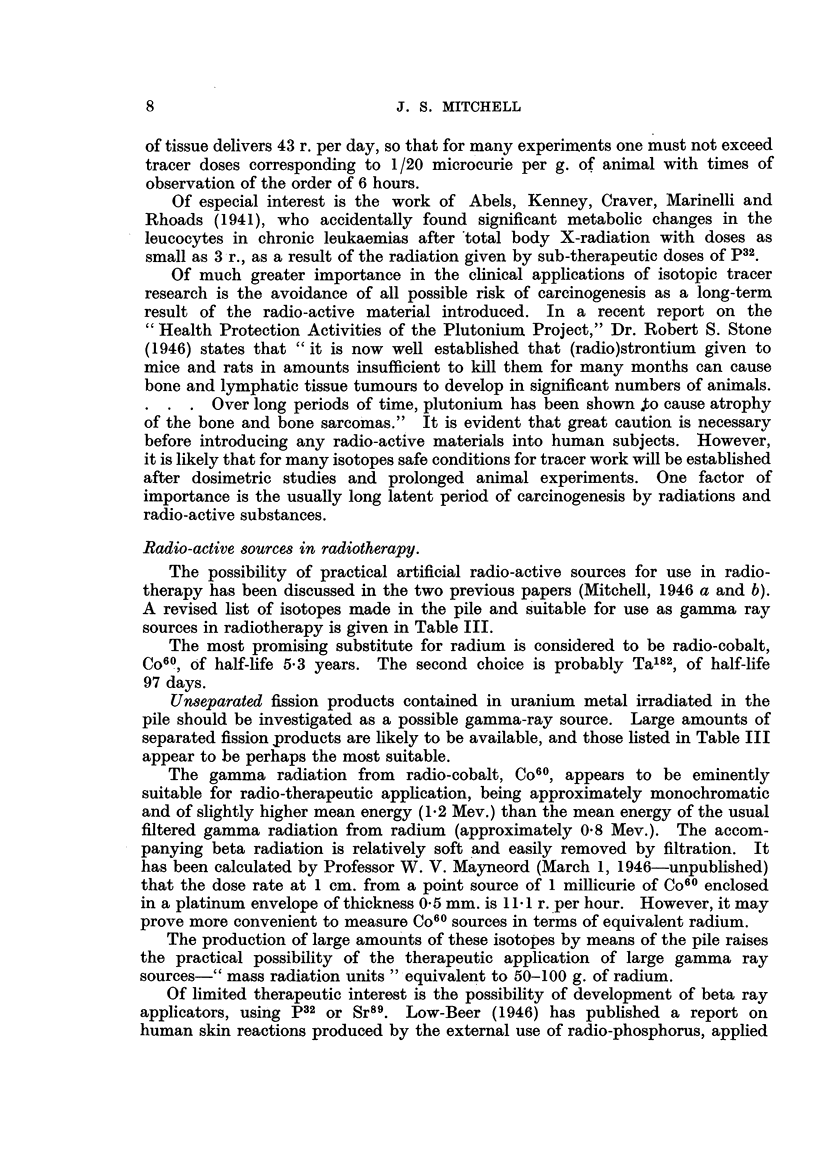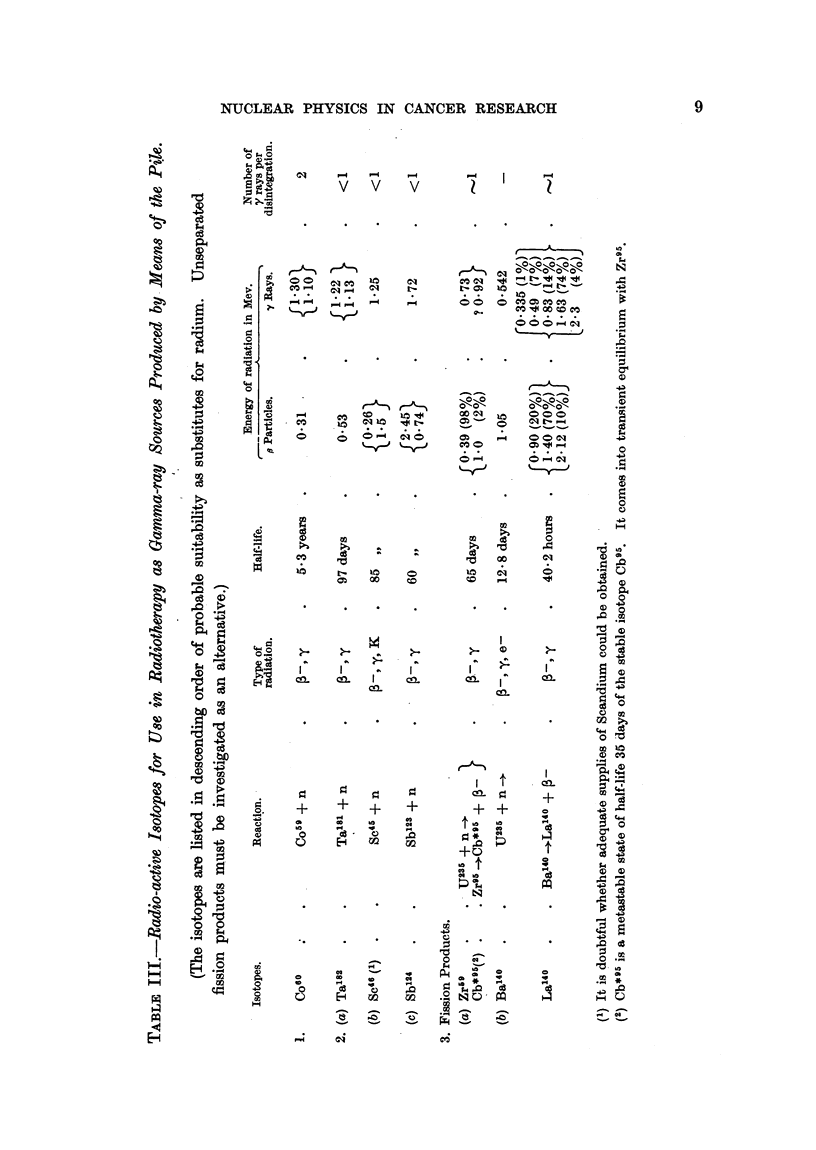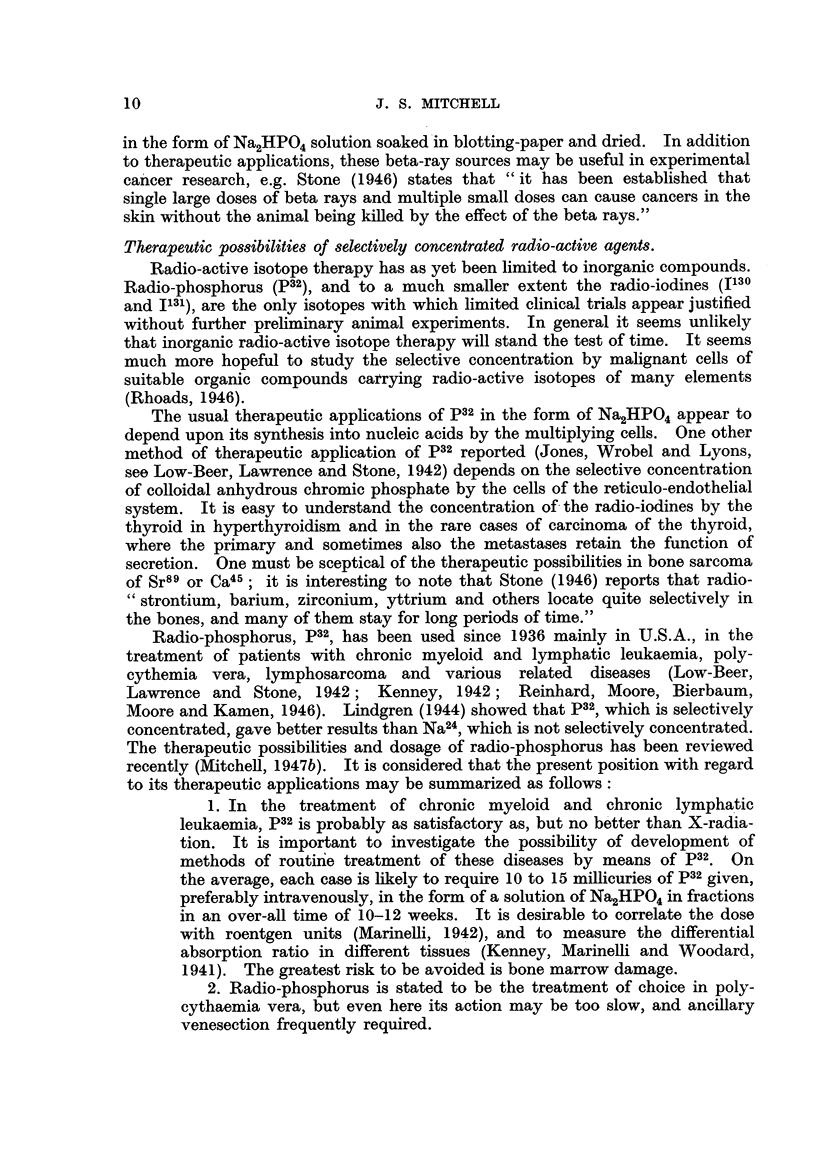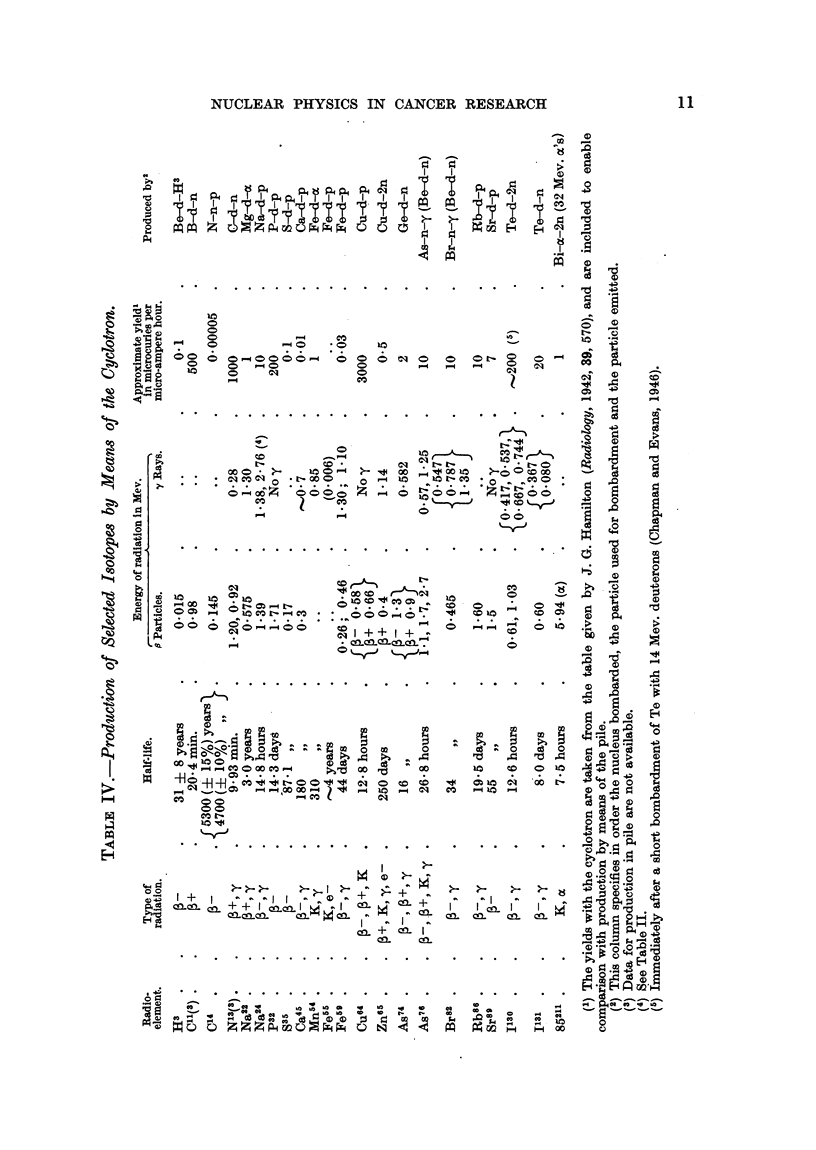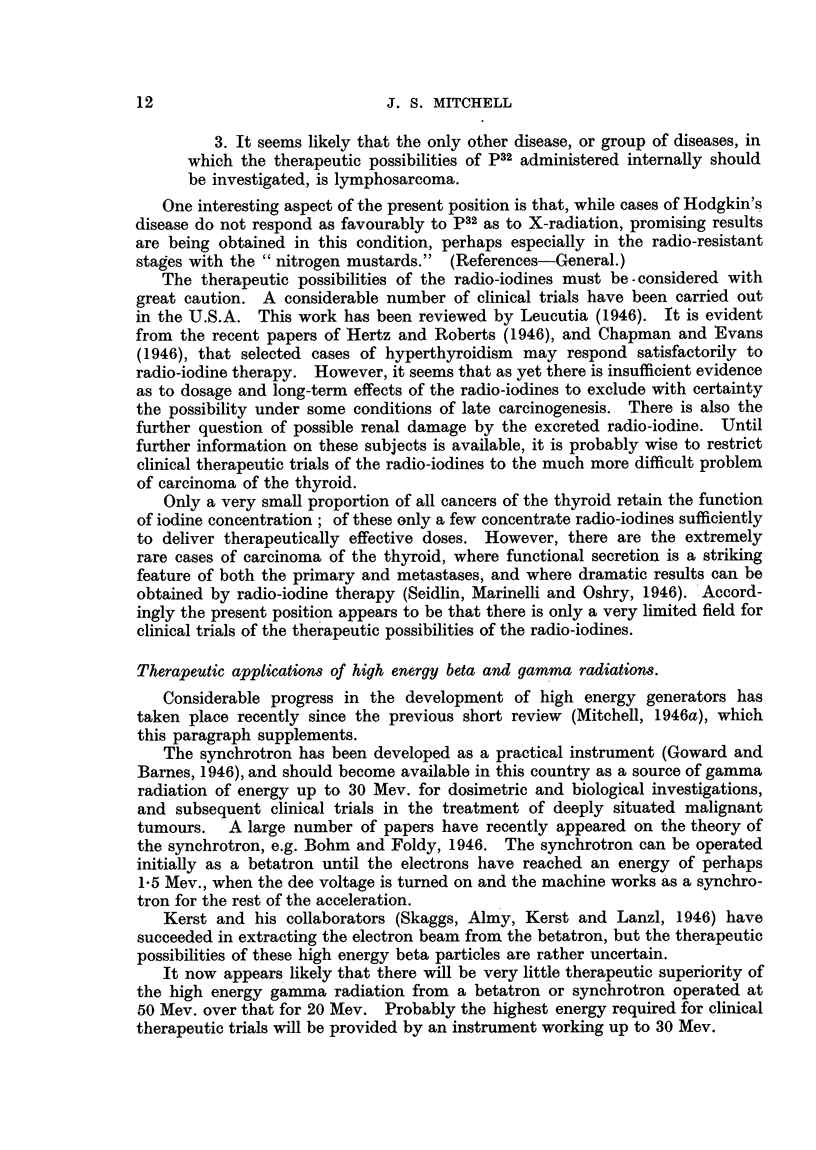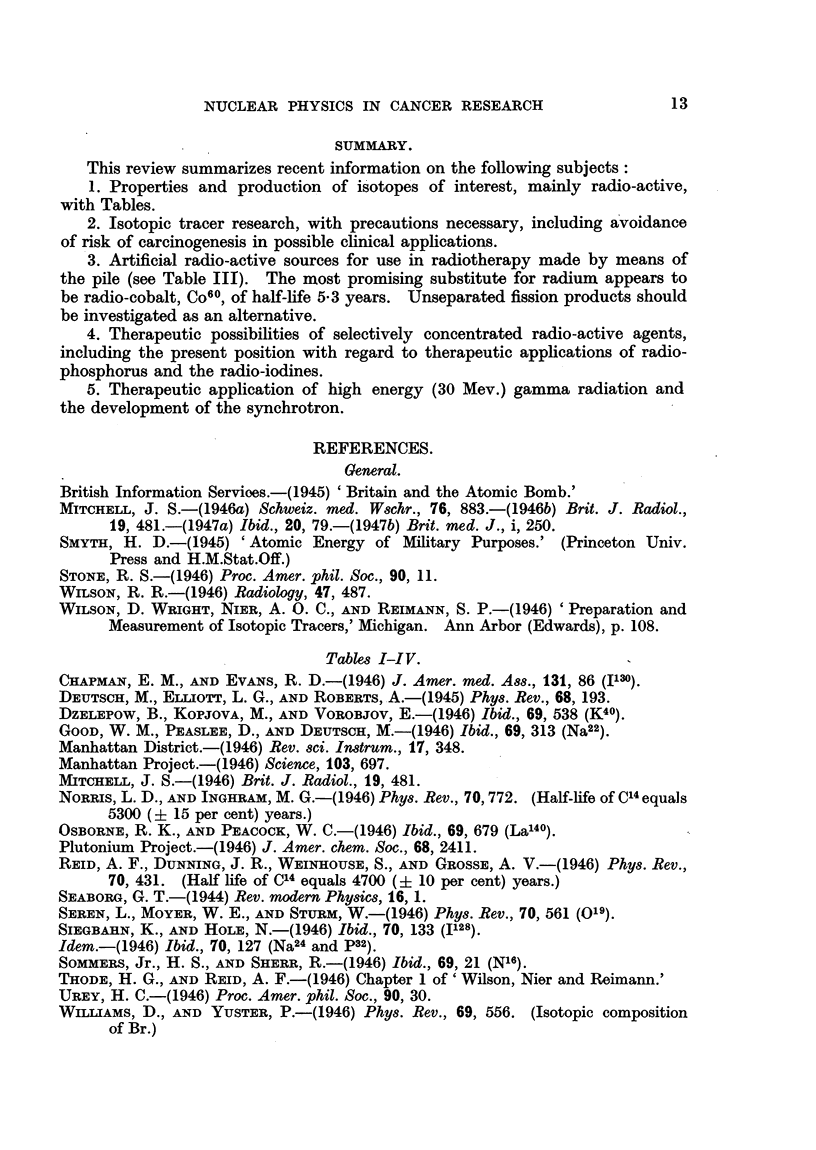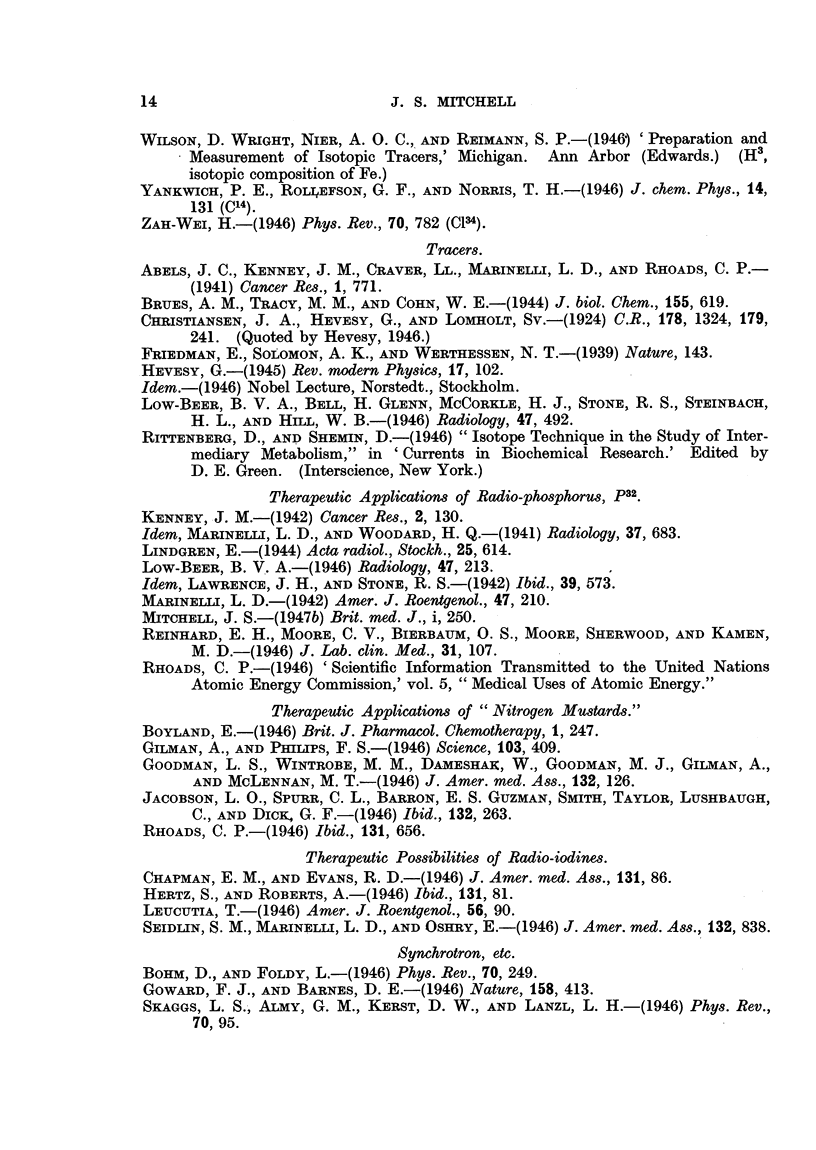# Applications of Recent Advances in Nuclear Physics to Cancer Research

**DOI:** 10.1038/bjc.1947.1

**Published:** 1947-03

**Authors:** J. S. Mitchell


BRITISH JOURNAL OF CANCER

VOL. I MARCH, 1947 NO. 1

### APPLICATIONS OF RECENT ADVANCES IN NUCLEAR PHYSICS TO CANCER RESEARCH.

J. S. MITCHELL.

*From Department of Radiotherapeutics, The University of Cambridge.*

Received for publication February 5, 1947.

THE immediate influence of the recent developments in nuclear physics upon the study of cancer is likely to be due to the provision of new and powerful tools for investigation and of new agents with therapeutic possibilities. It is more difficult to assess the significance of the studies of the mechanism of the biological action of radiations necessitated by the protection of large numbers of industrial workers from the radio-active hazards involved in making atomic bombs (R. S. Stone, 1946).

These subjects have been discussed in two previous papers (Mitchell, 1946, *a*, *b*). The present review includes more recent published information, and emphasizes different aspects.

The most important technical development is, of course, the chain-reacting "pile" (Smyth, 1945). This provides a method of preparation of very large amounts of many radio-active isotopes. It seems likely that these substances will ultimately become widely applied in medicine and biology as :

1. Tracers for the study of normal and pathological metabolic processes and in investigations in pharmacology and therapeutics.
2. Artificial radio-active sources mainly for use in radiotherapy as a substitute for radium.
3. Agents with therapeutic possibilities such as radio-phosphorus and the radio-iodines, and probably much better, suitable organic compounds containing radio-active isotopes, whose action depends on selective concentration of the radio-activity in particular cells.

The separation on the industrial scale of non-radio-active isotopes and the practical development of the mass spectrometer open new possibilities of isotopic tracer research where radio-active effects are undesirable, as in many clinical investigations, or where, as in the case of nitrogen and oxygen, no suitable radio-active isotopes exist.

The cyclotron still remains an essential instrument both for the production of small amounts of certain isotopes and as a source of fast neutrons for therapeutic trials in cancer and related diseases. It is by no means certain that the frequency modulated cyclotron will be useful as a therapeutic source of fast neutrons, on account of the possibility of reduced neutron output and of the undesirability of a pulsed output.

A promising field for radiotherapeutic investigation appears to be the study of high energy, 20–50 Mev. gamma radiation. The newly developed synchrotron appears to provide a practical source of high energy, e.g. 30 Mev. gamma radiation, much more easily obtainable than the betatron. It is possible that the linear accelerator may prove more suitable than the synchrotron. Although the electron beam can now be extracted from these machines, the therapeutic possibilities of the high energy beta particles are rather uncertain and must be investigated with caution.

The development of high energy generators opens the interesting possibility of the radiotherapeutic application of fast protons of energy in the region of 140 Mev. (Wilson, 1946).

The results of selected investigations are best summarized in the discussion of these newer agents.

### *Properties and Production of Isotopes, Radio-active and Stable.*

The properties of selected isotopes of especial interest are summarized in Table I. Recent information on isotopes, which may be useful as radio-active tracers, and which are produced in the pile, is summarized in Table II. A list of recent references on isotopes is also given.

The pile is without a rival for producing isotopes in the very large number of cases where, during irradiation in the pile, a simple slow neutron capture process occurs, e.g. $P^{31} + n \rightarrow P^{32}$, with emission of gamma radiation. Examples of specific activities obtainable by $n - \gamma$ reactions in the pile are for $P^{32}$ one-third curie per g. and for $S^{35}$ 10 millicuries per g.

There is one slow neutron (n — p) reaction of especial importance: $N^{14} + n \rightarrow C^{14} + p$. It is of interest that this process is probably responsible for 50–80 per cent of the biological effects of thermal neutrons, the remainder of the effects being due almost entirely to the $n - \gamma$ reaction in hydrogen (Mitchell, 1947). However, the n, p reaction in nitrogen is of extreme importance for isotopic tracer research as the basis for the preparation of $C^{14}$, the long-lived radio-active isotope of carbon. It has been shown by Yankwich, Rollefson and Norris (1946) that the $C^{14}$ is produced by slow neutron irradiation of compounds such as ammonium nitrate and urea in the form of very simple compounds such as $CO_2$,CO,$CH_3OH$,H.COOH, and of especial importance as a starting-point for organic syntheses, HCN.

In addition to the preparation of materials by direct irradiation, a very considerable number of radio-active isotopes are found as fission products in the uranium rods in a pile. A most valuable detailed list of these has been given by the Plutonium Project (1946).

In general, for the preparation of isotopes the yields with the cyclotron are very much smaller than those with the pile. A list of isotopes of interest made with the cyclotron is given in Table IV. The cyclotron can be used to prepare small amounts of *any* isotope. Further, the cyclotron is an essential supplement to the pile in order to produce—

(*a*) Alternative isotopes not made by slow neutron reactions, e.g. $Na^{22}$ (half-life 3·0 years), $Fe^{55}$ (half-life approximately 4 years).

(*b*) Higher concentrations of activity where preparation in the cyclotron involves a change in element and hence chemical separation is possible, e.g. $As^{74}$ (half-life 16 days).

(*c*) Short-lived isotopes, e.g. $C^{11}$ (half-life 20·4 mins.), $N^{13}$ (half-life 9·93 minutes), $F^{18}$ (half-life 112 minutes) and $I^{130}$ (half-life 12·6 hours), which is accompanied by smaller amounts of $I^{131}$ (half-life 8·0 days).

The methods used in the separation of stable isotopes have been reviewed by Thode and Reid (1946) and briefly by Urey (1946). The hydrogen isotopes, $H^1$ and $H^2$, have been separated on a large scale by distillation of water and the exchange reaction between water and hydrogen gas. The carbon isotopes have been partially separated by thermal diffusion and more cheaply by two chemical exchange reactions. The nitrogen isotopes have been separated by the exchange reaction between the ammonium ion in solution and ammonia gas and are now available commercially in U.S.A. The oxygen isotopes have been concentrated by distillation of water and by exchange reactions. Sulphur has been separated by chemical exchange between $SO_2$ and the bisulphite ion. Potassium has been also separated by chemical exchange methods.

The development of the mass-spectrometer as a practical instrument and its routine use for hydrocarbon analyses by industrial laboratories in U.S.A. show clearly the possibility in medicine and biology of non-radio-active isotopic tracer investigations. From this point of view, although deuterium still has its uses, the stable isotopes of greatest interest are $C^{13}$, $N^{15}$, $O^{18}$ and $S^{34}$.

A valuable little book entitled ‘ Preparation and Measurement of Isotopic Tracers ’ (edited by D. Wright Wilson (1946), J. W. Edwards, Ann Arbor), includes detailed accounts of the mass spectrometer and of the estimation of $C^{14}$, in addition to useful information about measurements with Geiger counters.

*Isotopic tracers.*

The first medical application of radio-active isotopic indicators appears to have been the investigation by Hevesy (Christiansen, Hevesy and Lomholt, 1924) of the absorption, distribution and excretion of bismuth in connection with the treatment of syphilis. Since then a surprisingly large literature of biochemical applications of stable and radio-active tracers has grown up. Interesting reviews have been published recently by Hevesy (1946) and Rittenberg and Shemin (1946).

Probably the most remarkable result of general biological importance emerging from tracer studies, mainly by Hevesy and Schoenheimer, is the discovery of the dynamic state of the tissue constituents. Almost all the constituent molecules of the animal body are continually broken down and resynthesized, so that the adult living cell is literally in a state of “ dynamic equilibrium.”

The most important application of tracers in radiotherapeutics so far has been the work of Hevesy (1945), who used radio-phosphorus ($P^{32}$) to demonstrate inhibition of synthesis of thymonucleic acid in normal and tumour cells by therapeutic doses of X-radiation, and the reality of indirect inhibiting effects on a second shielded tumour in the same animal. Another relevant application of $P^{32}$ is the work of Brues, Tracy and Cohn (1944), who showed that, while the turnover of thymonucleic acid phosphorus in non-growing liver is extremely slow, rapid synthesis of thymonucleic acid occurs in regenerative and neoplastic

TABLE I.—*Some Stable and Radio-active Isotopes of Medical and Biological Interest.*

| Element. | Isotope. | Stability or half-life. | Per cent abundance of stable isotope. | Type of radiation. | Energy of radiation in Mev. β Particles. | Energy of radiation in Mev. γ Rays. |
|---|---|---|---|---|---|---|
| Hydrogen | $H^1$ | Stable | 99·98 | .. | .. | .. |
| | $H^2$ | ,, | 0·02 | .. | .. | .. |
| | $H^3$ | 31 ± 8 yr. | .. | $\beta^-$ | 0·015 | .. |
| Carbon | $C^{11}$ | 20·4 min. | .. | $\beta^+$ | 0·98 | .. |
| | $C^{12}$ | Stable | 98·9 | .. | .. | .. |
| | $C^{13}$ | ,, | 1·1 | .. | .. | .. |
| | $C^{14}$ | ~5000 yr. | .. | $\beta^-$ | 0·145 | .. |
| Nitrogen | $N^{13}$ | 9·93 min. | .. | $\beta^+, \gamma$ | 0·92, 1·20 | 0·28 |
| | $N^{14}$ | Stable | 99·62 | .. | .. | .. |
| | $N^{15}$ | ,, | 0·38 | .. | .. | .. |
| | $N^{16}$ | 7·3 sec. | .. | $\beta^-$ | 10 ± 0·5 | .. |
| Oxygen | $O^{15}$ | 2·1 min. | .. | $\beta^+$ | 1·7 | .. |
| | $O^{16}$ | Stable | 99·76 | .. | .. | .. |
| | $O^{17}$ | ,, | 0·04 | .. | .. | .. |
| | $O^{18}$ | ,, | 0·20 | .. | .. | .. |
| | $O^{19}$ | 31 sec. | .. | $\beta^-$ | ~3·3 | .. |
| Fluorine | $F^{18}$ | 112 min. | .. | $\beta^+$ | 0·7 | .. |
| | $F^{19}$ | Stable | 100·0 | .. | .. | .. |
| Sodium | $Na^{22}$ | 3·0 yr. | .. | $\beta^+, \gamma$ | 0·575 | 1·30 |
| | $Na^{23}$ | Stable | 100·0 | .. | .. | .. |
| | $Na^{24}$ | 14·8 hr. | .. | $\beta^-, \gamma$ | 1·39 | 1·38, 2·76 |
| Magnesium | $Mg^{24}$ | Stable | 77·4 | .. | .. | .. |
| | $Mg^{25}$ | ,, | 11·5 | .. | .. | .. |
| | $Mg^{26}$ | ,, | 11·1 | .. | .. | .. |
| | $Mg^{27}$ | 10·2 min. | .. | $\beta^-, \gamma$ | 1·8 | 0·64, 0·84, 1·02 |
| Phosphorus | $P^{30}$ | 2·55 min. | .. | $\beta^+$ | 3·5 | .. |
| | $P^{31}$ | Stable | 100·0 | .. | .. | .. |
| | $P^{32}$ | 14·3 days | .. | $\beta^-$ | 1·71 | .. |
| Sulphur | $S^{32}$ | Stable | 95·1 | .. | .. | .. |
| | $S^{33}$ | ,, | 0·74 | .. | .. | .. |
| | $S^{34}$ | ,, | 4·2 | .. | .. | .. |
| | $S^{35}$ | 87·1 days | .. | $\beta^-$ | 0·17 | .. |
| | $S^{36}$ | Stable | 0·016 | .. | .. | .. |
| Chlorine | $Cl^{34}$ | 33 min. | .. | $\beta^+, \gamma$ | 2·4, 5·1 | ~3·4 |
| | $Cl^{35}$ | Stable | 75·4 | .. | .. | .. |
| | $Cl^{36}$ | ~$10^6$ yr. | .. | $\beta^-$ | 0·66 | .. |
| | $Cl^{37}$ | Stable | 24·6 | .. | .. | .. |
| | $Cl^{38}$ | 37 min. | .. | $\beta^-, \gamma$ | 1·1, 2·8, 5·0 | 1·65, 2·15 |
| Potassium | $K^{39}$ | Stable | 93·38 | .. | .. | .. |
| | $K^{40}$ | $1·4 \times 10^9$ yr. | 0·012 | $\beta^-, \gamma$ | 1·35 | ~2 |
| | $K^{41}$ | Stable | 6·61 | .. | .. | .. |
| | $K^{42}$ | 12·4 hr. | .. | $\beta^-$ | 3·5 | (?) |

TABLE I (continued).—*Some Stable and Radio-active Isotopes of Medical and Biological Interest.*

| Element. | Isotope. | Stability or half-life. | Per cent abundance of stable isotope. | Type of radiation. | Energy of radiation in Mev. | |
|---|---|---|---|---|---|---|
| | | | | | β Particles. | γ Rays. |
| Calcium | $Ca^{40}$ | Stable | 96·96 | .. | .. | .. |
| | $Ca^{41}$ | 8·5 days | .. | K, γ, e− | .. | 1·1 |
| | $Ca^{42}$ | Stable | 0·64 | .. | .. | .. |
| | $Ca^{43}$ | ,, | 0·15 | .. | .. | .. |
| | $Ca^{44}$ | ,, | 2·06 | .. | .. | .. |
| | $Ca^{45}$ | 180 days | .. | β−, γ | 0·3 | ~0·7 |
| | $Ca^{46}$ | Stable | 0·0033 | .. | .. | .. |
| | $Ca^{48}$ | ,, | 0·19 | .. | .. | .. |
| | $Ca^{49}$ | 2·5 hr. | .. | β−, γ | 2·3 | 0·8 |
| Iron | $Fe^{53}$ | 8·9 min. | .. | β+ | .. | .. |
| | $Fe^{54}$ | Stable | 5·9 | .. | .. | .. |
| | $Fe^{55}$ | ~ 4 yr. | .. | K, e− | .. | (0·006) |
| | $Fe^{56}$ | Stable | 91·7 | .. | .. | .. |
| | $Fe^{57}$ | ,, | 2·1 | .. | .. | .. |
| | $Fe^{58}$ | ,, | 0·3 | .. | .. | .. |
| | $Fe^{59}$ | 44 days | .. | β−, γ | 0·26, 0·46 | 1·30, 1·10 |
| Bromine | $Br^{79}$ | Stable | 50·6 | .. | .. | .. |
| | $Br^{80}$ | 4·4 hr. | .. | I.T.e−, γ | .. | 0·037 |
| | $Br^{81}$ | Stable | 49·4 | .. | .. | .. |
| | $Br^{82}$ | 34 hr. | .. | β−, γ | 0·465 | 0·547, 0·787, 1·35 |
| | $Br^{83}$ | 2·4 hr. | .. | β− | ~1·0 | .. |
| | $Br^{84}$ | 33 min. | .. | β−, γ | 5·3 | .. |
| Iodine | $I^{124}$ | 4·0 days | .. | β+ | .. | .. |
| | $I^{126}$ | 13 days | .. | β−, γ | ~1·1 | ~0·5 |
| | $I^{127}$ | Stable | 100 | .. | .. | .. |
| | $I^{128}$ | 25·0 min. | .. | β−, γ | 2·02 (93%) 1·59 (7%) | 0·428 |
| | $I^{129}$ | Very long | .. | β− | .. | .. |
| | $I^{130}$ | 12·6 hr. | .. | β−, γ | 0·61, 1·03 | 0·417, 0·537, 0·667, 0·744 |
| | $I^{131}$ | 8·0 days | .. | β−, γ, e− | 0·595 | 0·367, 0·080 |
| | $I^{133}$ | 22·0 hr. | .. | β−, γ | ~1·3 | ~0·55 |
| | $I^{135}$ | 6·7 hr. | .. | β−, γ | ~1·35 | ~1·6 |

β− = negative beta particle emitted from nucleus.

β+ = positive beta particle (positron).

γ = gamma ray.

e− = internal conversion electron.

I.T. = isomeric transition.

K = K-electron capture.

growth. An interesting possible diagnostic application of measurements of $P^{32}$ in breast tumours in situ has been reported by Low-Beer, Bell, McCorkle, Stone, Steinbach and Hill (1946).*

The application of $P^{32}$ as a tracer in metabolic investigations exemplifies the problems raised by this method of investigation. In order to avoid short-term effects, it is essential to calculate the dose of radiation received by the tissues from the radio-active isotope. For example, with $P^{32}$, one microcurie per g.

* One line of metabolic tracer investigation insufficiently well known is the use of radio-active brominated compounds, e.g. cholesterol dibromide (Friedman, Solomon and Werthessen, 1939).

of tissue delivers 43 r. per day, so that for many experiments one must not exceed tracer doses corresponding to 1/20 microcurie per g. of animal with times of observation of the order of 6 hours.

Of especial interest is the work of Abels, Kenney, Craver, Marinelli and Rhoads (1941), who accidentally found significant metabolic changes in the leucocytes in chronic leukaemias after total body X-radiation with doses as small as 3 r., as a result of the radiation given by sub-therapeutic doses of $P^{32}$.

Of much greater importance in the clinical applications of isotopic tracer research is the avoidance of all possible risk of carcinogenesis as a long-term result of the radio-active material introduced. In a recent report on the "Health Protection Activities of the Plutonium Project," Dr. Robert S. Stone (1946) states that "it is now well established that (radio)strontium given to mice and rats in amounts insufficient to kill them for many months can cause bone and lymphatic tissue tumours to develop in significant numbers of animals. . . . Over long periods of time, plutonium has been shown to cause atrophy of the bone and bone sarcomas." It is evident that great caution is necessary before introducing any radio-active materials into human subjects. However, it is likely that for many isotopes safe conditions for tracer work will be established after dosimetric studies and prolonged animal experiments. One factor of importance is the usually long latent period of carcinogenesis by radiations and radio-active substances.

*Radio-active sources in radiotherapy.*

The possibility of practical artificial radio-active sources for use in radiotherapy has been discussed in the two previous papers (Mitchell, 1946 *a* and *b*). A revised list of isotopes made in the pile and suitable for use as gamma ray sources in radiotherapy is given in Table III.

The most promising substitute for radium is considered to be radio-cobalt, $Co^{60}$, of half-life 5·3 years. The second choice is probably $Ta^{182}$, of half-life 97 days.

*Unseparated* fission products contained in uranium metal irradiated in the pile should be investigated as a possible gamma-ray source. Large amounts of separated fission products are likely to be available, and those listed in Table III appear to be perhaps the most suitable.

The gamma radiation from radio-cobalt, $Co^{60}$, appears to be eminently suitable for radio-therapeutic application, being approximately monochromatic and of slightly higher mean energy (1·2 Mev.) than the mean energy of the usual filtered gamma radiation from radium (approximately 0·8 Mev.). The accompanying beta radiation is relatively soft and easily removed by filtration. It has been calculated by Professor W. V. Mayneord (March 1, 1946—unpublished) that the dose rate at 1 cm. from a point source of 1 millicurie of $Co^{60}$ enclosed in a platinum envelope of thickness 0·5 mm. is 11·1 r. per hour. However, it may prove more convenient to measure $Co^{60}$ sources in terms of equivalent radium.

The production of large amounts of these isotopes by means of the pile raises the practical possibility of the therapeutic application of large gamma ray sources—"mass radiation units" equivalent to 50–100 g. of radium.

Of limited therapeutic interest is the possibility of development of beta ray applicators, using $P^{32}$ or $Sr^{89}$. Low-Beer (1946) has published a report on human skin reactions produced by the external use of radio-phosphorus, applied in the form of $Na_2HPO_4$ solution soaked in blotting-paper and dried. In addition to therapeutic applications, these beta-ray sources may be useful in experimental cancer research, e.g. Stone (1946) states that "it has been established that single large doses of beta rays and multiple small doses can cause cancers in the skin without the animal being killed by the effect of the beta rays."

*Therapeutic possibilities of selectively concentrated radio-active agents.*

Radio-active isotope therapy has as yet been limited to inorganic compounds. Radio-phosphorus ($P^{32}$), and to a much smaller extent the radio-iodines ($I^{130}$ and $I^{131}$), are the only isotopes with which limited clinical trials appear justified without further preliminary animal experiments. In general it seems unlikely that inorganic radio-active isotope therapy will stand the test of time. It seems much more hopeful to study the selective concentration by malignant cells of suitable organic compounds carrying radio-active isotopes of many elements (Rhoads, 1946).

The usual therapeutic applications of $P^{32}$ in the form of $Na_2HPO_4$ appear to depend upon its synthesis into nucleic acids by the multiplying cells. One other method of therapeutic application of $P^{32}$ reported (Jones, Wrobel and Lyons, see Low-Beer, Lawrence and Stone, 1942) depends on the selective concentration of colloidal anhydrous chromic phosphate by the cells of the reticulo-endothelial system. It is easy to understand the concentration of the radio-iodines by the thyroid in hyperthyroidism and in the rare cases of carcinoma of the thyroid, where the primary and sometimes also the metastases retain the function of secretion. One must be sceptical of the therapeutic possibilities in bone sarcoma of $Sr^{89}$ or $Ca^{45}$; it is interesting to note that Stone (1946) reports that radio-"strontium, barium, zirconium, yttrium and others locate quite selectively in the bones, and many of them stay for long periods of time."

Radio-phosphorus, $P^{32}$, has been used since 1936 mainly in U.S.A., in the treatment of patients with chronic myeloid and lymphatic leukaemia, polycythemia vera, lymphosarcoma and various related diseases (Low-Beer, Lawrence and Stone, 1942; Kenney, 1942; Reinhard, Moore, Bierbaum, Moore and Kamen, 1946). Lindgren (1944) showed that $P^{32}$, which is selectively concentrated, gave better results than $Na^{24}$, which is not selectively concentrated. The therapeutic possibilities and dosage of radio-phosphorus has been reviewed recently (Mitchell, 1947*b*). It is considered that the present position with regard to its therapeutic applications may be summarized as follows:

1. In the treatment of chronic myeloid and chronic lymphatic leukaemia, $P^{32}$ is probably as satisfactory as, but no better than X-radiation. It is important to investigate the possibility of development of methods of routine treatment of these diseases by means of $P^{32}$. On the average, each case is likely to require 10 to 15 millicuries of $P^{32}$ given, preferably intravenously, in the form of a solution of $Na_2HPO_4$ in fractions in an over-all time of 10–12 weeks. It is desirable to correlate the dose with roentgen units (Marinelli, 1942), and to measure the differential absorption ratio in different tissues (Kenney, Marinelli and Woodard, 1941). The greatest risk to be avoided is bone marrow damage.

2. Radio-phosphorus is stated to be the treatment of choice in polycythaemia vera, but even here its action may be too slow, and ancillary venesection frequently required.

(4) See Table II.

(5) Immediately after a short bombardment of Te with 14 Mev. deuterons (Chapman and Evans, 1946).

3. It seems likely that the only other disease, or group of diseases, in which the therapeutic possibilities of $P^{32}$ administered internally should be investigated, is lymphosarcoma.

One interesting aspect of the present position is that, while cases of Hodgkin's disease do not respond as favourably to $P^{32}$ as to X-radiation, promising results are being obtained in this condition, perhaps especially in the radio-resistant stages with the "nitrogen mustards." (References—General.)

The therapeutic possibilities of the radio-iodines must be considered with great caution. A considerable number of clinical trials have been carried out in the U.S.A. This work has been reviewed by Leucutia (1946). It is evident from the recent papers of Hertz and Roberts (1946), and Chapman and Evans (1946), that selected cases of hyperthyroidism may respond satisfactorily to radio-iodine therapy. However, it seems that as yet there is insufficient evidence as to dosage and long-term effects of the radio-iodines to exclude with certainty the possibility under some conditions of late carcinogenesis. There is also the further question of possible renal damage by the excreted radio-iodine. Until further information on these subjects is available, it is probably wise to restrict clinical therapeutic trials of the radio-iodines to the much more difficult problem of carcinoma of the thyroid.

Only a very small proportion of all cancers of the thyroid retain the function of iodine concentration ; of these only a few concentrate radio-iodines sufficiently to deliver therapeutically effective doses. However, there are the extremely rare cases of carcinoma of the thyroid, where functional secretion is a striking feature of both the primary and metastases, and where dramatic results can be obtained by radio-iodine therapy (Seidlin, Marinelli and Oshry, 1946). Accordingly the present position appears to be that there is only a very limited field for clinical trials of the therapeutic possibilities of the radio-iodines.

*Therapeutic applications of high energy beta and gamma radiations.*

Considerable progress in the development of high energy generators has taken place recently since the previous short review (Mitchell, 1946*a*), which this paragraph supplements.

The synchrotron has been developed as a practical instrument (Goward and Barnes, 1946), and should become available in this country as a source of gamma radiation of energy up to 30 Mev. for dosimetric and biological investigations, and subsequent clinical trials in the treatment of deeply situated malignant tumours. A large number of papers have recently appeared on the theory of the synchrotron, e.g. Bohm and Foldy, 1946. The synchrotron can be operated initially as a betatron until the electrons have reached an energy of perhaps 1·5 Mev., when the dee voltage is turned on and the machine works as a synchrotron for the rest of the acceleration.

Kerst and his collaborators (Skaggs, Almy, Kerst and Lanzl, 1946) have succeeded in extracting the electron beam from the betatron, but the therapeutic possibilities of these high energy beta particles are rather uncertain.

It now appears likely that there will be very little therapeutic superiority of the high energy gamma radiation from a betatron or synchrotron operated at 50 Mev. over that for 20 Mev. Probably the highest energy required for clinical therapeutic trials will be provided by an instrument working up to 30 Mev.

SUMMARY.

This review summarizes recent information on the following subjects :

1. Properties and production of isotopes of interest, mainly radio-active, with Tables.

2. Isotopic tracer research, with precautions necessary, including avoidance of risk of carcinogenesis in possible clinical applications.

3. Artificial radio-active sources for use in radiotherapy made by means of the pile (see Table III). The most promising substitute for radium appears to be radio-cobalt, $Co^{60}$, of half-life 5·3 years. Unseparated fission products should be investigated as an alternative.

4. Therapeutic possibilities of selectively concentrated radio-active agents, including the present position with regard to therapeutic applications of radio-phosphorus and the radio-iodines.

5. Therapeutic application of high energy (30 Mev.) gamma radiation and the development of the synchrotron.

### REFERENCES.

### *General.*

British Information Services.—(1945) ' Britain and the Atomic Bomb.'

MITCHELL, J. S.—(1946*a*) *Schweiz. med. Wschr.*, **76**, 883.—(1946*b*) *Brit. J. Radiol.*, **19**, 481.—(1947*a*) *Ibid.*, **20**, 79.—(1947*b*) *Brit. med. J.*, i, 250.

SMYTH, H. D.—(1945) ' Atomic Energy of Military Purposes.' (Princeton Univ. Press and H.M.Stat.Off.)

STONE, R. S.—(1946) *Proc. Amer. phil. Soc.*, **90**, 11.

WILSON, R. R.—(1946) *Radiology*, **47**, 487.

WILSON, D. WRIGHT, NIER, A. O. C., AND REIMANN, S. P.—(1946) ' Preparation and Measurement of Isotopic Tracers,' Michigan. Ann Arbor (Edwards), p. 108.

### *Tables I–IV.*

CHAPMAN, E. M., AND EVANS, R. D.—(1946) *J. Amer. med. Ass.*, **131**, 86 ($I^{130}$).

DEUTSCH, M., ELLIOTT, L. G., AND ROBERTS, A.—(1945) *Phys. Rev.*, **68**, 193.

DZELEPOW, B., KOPJOVA, M., AND VOROBJOV, E.—(1946) *Ibid.*, **69**, 538 ($K^{40}$).

GOOD, W. M., PEASLEE, D., AND DEUTSCH, M.—(1946) *Ibid.*, **69**, 313 ($Na^{22}$).

Manhattan District.—(1946) *Rev. sci. Instrum.*, **17**, 348.

Manhattan Project.—(1946) *Science*, **103**, 697.

MITCHELL, J. S.—(1946) *Brit. J. Radiol.*, **19**, 481.

NORRIS, L. D., AND INGHRAM, M. G.—(1946) *Phys. Rev.*, **70**, 772. (Half-life of $C^{14}$ equals 5300 (± 15 per cent) years.)

OSBORNE, R. K., AND PEACOCK, W. C.—(1946) *Ibid.*, **69**, 679 ($La^{140}$).

Plutonium Project.—(1946) *J. Amer. chem. Soc.*, **68**, 2411.

REID, A. F., DUNNING, J. R., WEINHOUSE, S., AND GROSSE, A. V.—(1946) *Phys. Rev.*, **70**, 431. (Half life of $C^{14}$ equals 4700 (± 10 per cent) years.)

SEABORG, G. T.—(1944) *Rev. modern Physics*, **16**, 1.

SEREN, L., MOYER, W. E., AND STURM, W.—(1946) *Phys. Rev.*, **70**, 561 ($O^{19}$).

SIEGBAHN, K., AND HOLE, N.—(1946) *Ibid.*, **70**, 133 ($I^{128}$).

*Idem.*—(1946) *Ibid.*, **70**, 127 ($Na^{24}$ and $P^{32}$).

SOMMERS, Jr., H. S., AND SHERR, R.—(1946) *Ibid.*, **69**, 21 ($N^{16}$).

THODE, H. G., AND REID, A. F.—(1946) Chapter 1 of ' Wilson, Nier and Reimann.'

UREY, H. C.—(1946) *Proc. Amer. phil. Soc.*, **90**, 30.

WILLIAMS, D., AND YUSTER, P.—(1946) *Phys. Rev.*, **69**, 556. (Isotopic composition of Br.)

WILSON, D. WRIGHT, NIER, A. O. C., AND REIMANN, S. P.—(1946) 'Preparation and Measurement of Isotopic Tracers,' Michigan. Ann Arbor (Edwards.) ($H^3$, isotopic composition of Fe.)
YANKWICH, P. E., ROLLEFSON, G. F., AND NORRIS, T. H.—(1946) *J. chem. Phys.*, **14**, 131 ($C^{14}$).
ZAH-WEI, H.—(1946) *Phys. Rev.*, **70**, 782 ($Cl^{34}$).

*Tracers.*

ABELS, J. C., KENNEY, J. M., CRAVER, LL., MARINELLI, L. D., AND RHOADS, C. P.—(1941) *Cancer Res.*, **1**, 771.
BRUES, A. M., TRACY, M. M., AND COHN, W. E.—(1944) *J. biol. Chem.*, **155**, 619.
CHRISTIANSEN, J. A., HEVESY, G., AND LOMHOLT, SV.—(1924) *C.R.*, **178**, 1324, **179**, 241. (Quoted by Hevesy, 1946.)
FRIEDMAN, E., SOLOMON, A. K., AND WERTHESSEN, N. T.—(1939) *Nature*, 143.
HEVESY, G.—(1945) *Rev. modern Physics*, **17**, 102.
*Idem.*—(1946) Nobel Lecture, Norstedt., Stockholm.
LOW-BEER, B. V. A., BELL, H. GLENN, MCCORKLE, H. J., STONE, R. S., STEINBACH, H. L., AND HILL, W. B.—(1946) *Radiology*, **47**, 492.
RITTENBERG, D., AND SHEMIN, D.—(1946) " Isotope Technique in the Study of Intermediary Metabolism," in 'Currents in Biochemical Research.' Edited by D. E. Green. (Interscience, New York.)

*Therapeutic Applications of Radio-phosphorus, $P^{32}$.*

KENNEY, J. M.—(1942) *Cancer Res.*, **2**, 130.
*Idem*, MARINELLI, L. D., AND WOODARD, H. Q.—(1941) *Radiology*, **37**, 683.
LINDGREN, E.—(1944) *Acta radiol., Stockh.*, **25**, 614.
LOW-BEER, B. V. A.—(1946) *Radiology*, **47**, 213.
*Idem*, LAWRENCE, J. H., AND STONE, R. S.—(1942) *Ibid.*, **39**, 573.
MARINELLI, L. D.—(1942) *Amer. J. Roentgenol.*, **47**, 210.
MITCHELL, J. S.—(1947*b*) *Brit. med. J.*, i, 250.
REINHARD, E. H., MOORE, C. V., BIERBAUM, O. S., MOORE, SHERWOOD, AND KAMEN, M. D.—(1946) *J. Lab. clin. Med.*, **31**, 107.
RHOADS, C. P.—(1946) 'Scientific Information Transmitted to the United Nations Atomic Energy Commission,' vol. 5, " Medical Uses of Atomic Energy."

*Therapeutic Applications of " Nitrogen Mustards."*

BOYLAND, E.—(1946) *Brit. J. Pharmacol. Chemotherapy*, **1**, 247.
GILMAN, A., AND PHILIPS, F. S.—(1946) *Science*, **103**, 409.
GOODMAN, L. S., WINTROBE, M. M., DAMESHAK, W., GOODMAN, M. J., GILMAN, A., AND MCLENNAN, M. T.—(1946) *J. Amer. med. Ass.*, **132**, 126.
JACOBSON, L. O., SPURR, C. L., BARRON, E. S. GUZMAN, SMITH, TAYLOR, LUSHBAUGH, C., AND DICK, G. F.—(1946) *Ibid.*, **132**, 263.
RHOADS, C. P.—(1946) *Ibid.*, **131**, 656.

*Therapeutic Possibilities of Radio-iodines.*

CHAPMAN, E. M., AND EVANS, R. D.—(1946) *J. Amer. med. Ass.*, **131**, 86.
HERTZ, S., AND ROBERTS, A.—(1946) *Ibid.*, **131**, 81.
LEUCUTIA, T.—(1946) *Amer. J. Roentgenol.*, **56**, 90.
SEIDLIN, S. M., MARINELLI, L. D., AND OSHRY, E.—(1946) *J. Amer. med. Ass.*, **132**, 838.

*Synchrotron, etc.*

BOHM, D., AND FOLDY, L.—(1946) *Phys. Rev.*, **70**, 249.
GOWARD, F. J., AND BARNES, D. E.—(1946) *Nature*, **158**, 413.
SKAGGS, L. S., ALMY, G. M., KERST, D. W., AND LANZL, L. H.—(1946) *Phys. Rev.*, **70**, 95.